# Supplementary material for: Investigating medication adherence among Taiwanese patient with hypertension, hyperlipidemia, and diabetes: A pilot study using the Chinese version of a Two-Part Medication Nonadherence Scale and the NHI MediCloud system
Source: PLoS One. 2024 Jul 10;19(7):e0304442. doi: 10.1371/journal.pone.0304442 (PMC11236195; doi:10.1371/journal.pone.0304442)
Supplement: S1 Appendix — (DOCX) [file pone.0304442.s001.docx]

**Appendix I.: Chinese Two-Part Medication Nonadherence Scale (C-TPMNS)** Date: _____ month ___ day number:

**Basic personal data**

1. What is your actual date of birth?

□ ___ month ___ day, _____ year of the Republic of China (□ National calendar □ Lunar calendar)

□ I don’t know the actual date of birth, but I know my age: I am now 10 years old

□ Not sure

2.Your gender is: □ Male □ Female

3. Your height is: ＿＿cm; weight is: ＿＿＿kg

4.Your occupation is:

□ Never had a formal job

□ Currently retired

□ Student □ Military, public and educational □ Agriculture □ Fishing □ Industry □ Business □ Other (occupation is: ________)

5. What is your highest educational level? (subject to graduation)

□ Illiterate □ Elementary school □ Didn’t graduate from elementary school □ Junior high school (vocational) □ High school (vocational) □ College

Studies (second or third college, second or fourth technical college) □ graduate school and above

6. What is your marital status?

□ Single □ Married/cohabiting □ Divorced/separated □ Widow

7.Who do you live with?

□ Living alone □ Living with family □ Living with friends □ Living in long-term care facility □ Other______

8. What is your average monthly income?

□ Less than 20,000 NT dollar □ More than 20,000 NT dollar ~ less than 30,000 NT dollar □ More than 30,000 NT dollar ~ less than

Over 50,000 NT dollar □ More than 50,000 NT dollar

9. Do you have diabetes? □ Yes, have been ill for ___ years □ No

10.Do you have high blood pressure? □ Yes, have been ill for ___ years □ No

11. Do you have high blood lipids? □ Yes, have been ill for ___ years □ No

12.Have you ever seen any advertisements or promotions related to drug recycling? □ Yes (Where did you get it? Can you check?

□ TV □ Newspapers □ Online news and publicity □ Social software forwarding □ Relatives and friends □ Medical institutions)

□ No

13. Do you know where there is a drug recycling service? □ Yes (please check the recycling location, you can

Check □ pharmacy, □ hospital, □ health center) □ No

14. Do you have any experience in drug recycling? □ Yes ( times/year) □ No

15. Do you have any experience with leftover expired medicines or leftover medicines? □ Yes □ No

16.Which type of drugs have you ever recycled (optional)? □ Anti-cancer and immunosuppressive drugs antibiotics

□ Controlled drugs (such as sedatives, sleeping pills) □ Hormones □ Discarded needles and needles □ Three

High Drugs □ Others＿＿

17. Are you here to recycle medicines this time? □ Yes (Number of recycled pills □ 0~100 pills □ 100~200

□ 200~500 □ More than 500 □ No

**Health insurance medical information cloud inquiry system record data**

18. Please check the following respondent’s disease history:

□ Complications of diabetes (nephropathy, eye disease, peripheral neuropathy, diabetic foot, amputation, etc.)

□ Depression □ Cancer □ Kidney disease □ Rheumatoid arthritis □ Others＿＿＿＿(if no disease

Sick, please write None)

19. How many medications does the respondent take at one time (use the most recent information):

□ 1 type □ 2 types □ 3 types □ 4 types □ 5 or more types

20. The number of medical visits made by the respondent in the past three months: outpatient visits, emergency room visits, hospitalization ____ times

21. The respondent’s three high test values ​​in the past six months (take the most recent one): Glycated hemoglobin test value

_________, blood sugar level before meal, blood sugar level after meal; systolic blood pressure,Diastolic blood pressure; total cholesterol, low-density lipoprotein cholesterol_______; triglycerides. _______

22. Please fill in the following drug information

|  | hypoglycemic drugs | Treatment of High Blood Pressure | hypolipidemic drugs | triglyceride-lowering medications | others |
| --- | --- | --- | --- | --- | --- |
| Types of drugs prescribed Types of medicines to be recycled to pharmacies | ○ | ○ | ○ | ○ | ○ |
| Types of medicines to be recycled to pharmaciesmedicine | ○ | ○ | ○ | ○ | ○ |

23. Please fill in the following information to estimate the Medication Possession Rate (MPR) in the past six months

|  | hypoglycemic drugs | Treatment of High Blood Pressure | hypolipidemic drugs |
| --- | --- | --- | --- |
| Date of first prescription | ○ | ○ | ○ |
| Date of last prescription | ○ | ○ | ○ |
| Number of days since last prescription |  |  |  |
| The total number of actual days of receiving medication |  |  |  |
