# Supplementary material for: Investigating medication adherence among Taiwanese patient with hypertension, hyperlipidemia, and diabetes: A pilot study using the Chinese version of a Two-Part Medication Nonadherence Scale and the NHI MediCloud system
Source: PLoS One. 2024 Jul 10;19(7):e0304442. doi: 10.1371/journal.pone.0304442 (PMC11236195; doi:10.1371/journal.pone.0304442)
Supplement: S1 File — (DOC) [file pone.0304442.s002.doc]

Thank you for participating in this study. This consent form is provided to give you relevant information about the study so that you can decide whether to participate. The principal investigator or designated research personnel will explain the details of the study and answer any questions you may have. You are free to ask any questions related to this study, and please do not sign this consent form until your questions have been satisfactorily addressed. If you choose to participate in this study, this document will serve as a record of your consent. Even after giving your consent, you have the right to withdraw from the study at any time without providing any reason.

| **Research Project Title**  **Non-adherence to Glucose-, Blood Pressure-, and Lipid-lowering Drugs and Associated Factors** | |
| --- | --- |
| **Research Project Overview**  Research Objectives: This project aims to understand the extent and types of medication non-adherence among patients with three high-risk factors (hypertension, hyperlipidemia, and hyperglycemia). The study will analyze factors related to non-adherence and provide specific recommendations for improving medication adherence in patients with these three high-risk conditions.  Research Methods and Procedures:  Research personnel will collect data through two questionnaires, each taking approximately 10 to 15 minutes to complete. The first questionnaire, consisting of about 15 questions, includes basic information, medical history, and records of prescribed medications and relevant tests for the three high-risk conditions. Some data, such as medication prescription records and relevant test results, will be collected through the National Health Insurance medical information cloud query system. The second questionnaire is a medication adherence scale comprising 21 questions.  Research Project Timeline: The project will be executed from July 1, 2019, to July 31, 2021.  Recruitment Criteria for Research Participants:  (1) Inclusion Criteria: Individuals aged 20 and above who obtain medications for the three high-risk conditions from chain pharmacies in the central region or those who return expired medications for these conditions to pharmacies for disposal. Additional participants without these conditions will be recruited as a control group.  (2) Exclusion Criteria: Individuals diagnosed with chronic conditions, including the three high-risk factors, within the six months leading up to the data collection date. |  |
| **Anticipated Risks and Disposal Methods for Participation in the Study**  Discomfort or Distress: If any questions or discussions during the study make you feel uncomfortable or distressed, you have the right to refuse to answer any questions or withdraw from the discussion at any time.  Privacy Concerns: This research project will not collect or record personal identifiable information. The principal investigator of this project will be responsible for the storage of research data, and the data will not be provided to others outside the research team. Your provided information will be treated with confidentiality.  Data Security Measures: All collected data will be securely stored by the principal investigator, and access will be restricted to the research team. Personal identifiers will be removed from the data to ensure anonymity. The research team is committed to maintaining the confidentiality and privacy of the information you provide.  Withdrawal Option: You have the right to withdraw from the study at any time without facing any negative consequences or providing a reason for your decision.  Informed Decision-Making: Before participating in the study, you have been provided with relevant information and have the opportunity to ask questions. Your decision to participate is voluntary, and you are encouraged to make an informed decision based on your understanding of the study's purpose, procedures, and potential risks.  By participating in this study, it is understood that you have read and understood the potential risks involved and agree to the measures outlined to address these risks. |  |
| **Confidentiality** This project will not record personally identifiable information that can identify you. If research personnel come across personal privacy information during the research process, it will not be disclosed to the public or shared with individuals not involved in this study. Researchers, members of the research ethics committee, and the sponsoring organization all have legal rights to review your research records to ensure the appropriateness and adequate protection of your rights as a research participant. However, under normal circumstances, only the research personnel of this project will have access to identifiable information about you. Unless necessary, researchers will not report this information to other entities, and all personnel mentioned above commit not to violate the confidentiality of your identity.  This project does not record your name. If the research results are published, your identity will remain confidential. Your responses will be coded, and all research records will be securely stored and locked in a file cabinet in the project leader's office. The retention period is three years after the completion of the research, and the records will be destroyed at the end of this period. |  |
| If you experience adverse reactions as a result of participating in this project, the project is willing to provide free professional psychological counseling/medical services. Please contact Miss Zhang Yinxuan at 0972-500761 for assistance. However, compensation will not be provided for anticipated adverse reactions as stated in the research participant consent form. |  |
| **Compensation**  If you experience harm due to adverse reactions from participating in this project, China Medical University will assume legal responsibility for compensation. Signing this consent form does not waive any of your legal rights. | |
| - **Participation, Suspension, and Withdrawal from the Study** - You are free to decide whether to participate in this project. During the research process, you have the right to withdraw your consent or exit the study at any time without providing any reason. Your decision to withdraw will not result in any discomfort, additional penalties, or impact on any other aspects of your rights (e.g., employment/medical care/school grades). | |
| **Contact Information**  　　If you have any concerns or complaints about the execution of this project, the procedures adopted, the risks and benefits, or your rights as a research participant, please contact the Research Ethics Committee of China Medical University and Hospital at the following: Phone: 04-22052121 ext. 1941, 1923~1927, 1929  E-mail: rrec@mail.cmu.edu.tw or irb@mail.cmuh.org.tw  Subject: [Inquiry or Complaint from Research Participant] | |
| - 1. signature   (二) The researcher has provided detailed explanations regarding the nature and objectives of the research methods outlined in this research project, as well as the potential risks and benefits.  Signature of the Research Explainer: ________ (Signature)  Signature Date:： year Month Day  The research participant has thoroughly understood the aforementioned research methods and the potential risks and benefits associated with them. Any questions regarding this research project have been adequately explained by the principal investigator or the designated research explainer. I voluntarily consent to participate in this research project.  Participant's Full Name: _______________ Signature: ______________  Signature Date:： year Month Day | |
|  | |
