# Supplementary material for: Investigating medication adherence among Taiwanese patient with hypertension, hyperlipidemia, and diabetes: A pilot study using the Chinese version of a Two-Part Medication Nonadherence Scale and the NHI MediCloud system
Source: PLoS One. 2024 Jul 10;19(7):e0304442. doi: 10.1371/journal.pone.0304442 (PMC11236195; doi:10.1371/journal.pone.0304442)
Supplement: Attachment — Submitted filename: 20240401_response to reviewer.docx [file pone.0304442.s003.docx]

**Response to Reviewers’ comments**

**Manuscript ID:** [PONE-D-23-38644] - [EMID:ea540dbfb190153a]

**MS TITLE:** Investigating Patient Adherence to Medications for Hypertension, Hyperlipidemia, and Diabetes: A Study on Expired Medication Recycling at Community Pharmacies Using the Chinese Version of a Two-Part Medication Nonadherence Scale

**Response to Editor**

**Editor Comments to the Author**

*We've checked your submission and before we can proceed, we need you to address the following issues:*

*1. In the online submission form, you indicated that your data is available only on request from a third party. Please note that your Data Availability Statement is currently missing the name of the third party contact or institution / contact details for the third party, such as an email address or a link to where data requests can be made. Please update your statement with the missing information.*

Reply: Thank you for reminding. We have added contact details for the third party in the Data Availability section. We have revised as “The raw data involved in this analysis are available. The collected questionnaires were stored and locked in the office to ensure data confidentiality. Only participants ID codes were used throughout the study. Data were not used by a third party. For further access to the database, please contact the Ministry of Health and Welfare (Email: stcarolwu@mohw.gov.tw) for further assistance. Taiwan Ministry of Health and Welfare Address: 488 Zhongxiao E. Rd. Sec. 6, Nangang Dist., Taipei 115, Taiwan (R.O.C.). Phone: +886-2-8590-6848). Participation in the study was entirely voluntary, and anonymity was highly guaranteed. (Please see the revised manuscript.)
